# Hypothalamic Vasopressinergic Projections Innervate Central Amygdala GABAergic Neurons: Implications for Anxiety and Stress Coping

**DOI:** 10.3389/fncir.2016.00092

**Published:** 2016-11-18

**Authors:** Vito S. Hernández, Oscar R. Hernández, Miguel Perez de la Mora, María J. Gómora, Kjell Fuxe, Lee E. Eiden, Limei Zhang

**Affiliations:** ^1^Departamento de Fisiología, Facultad de Medicina, Universidad Nacional Autónoma de MéxicoMexico City, Mexico; ^2^División de Neurociencias, Instituto de Fisiología Celular, Universidad Nacional Autónoma de MéxicoMexico City, Mexico; ^3^Departamento de Embriología, Facultad de Medicina, Universidad Nacional Autónoma de MéxicoMexico City, Mexico; ^4^Department of Neuroscience, Karolinska InstitutetStockholm, Sweden; ^5^Section on Molecular Neuroscience, National Institute of Mental Health, National Institutes of Health (NIH)Bethesda, MD, USA

**Keywords:** magnocellular, synapses, RNAscope, Gad1, Gad2, V1a, microinjection, maternal separation

## Abstract

The arginine-vasopressin (AVP)-containing hypothalamic magnocellular neurosecretory neurons (VPMNNs) are known for their role in hydro-electrolytic balance control via their projections to the neurohypophysis. Recently, projections from these same neurons to hippocampus, habenula and other brain regions in which vasopressin infusion modulates contingent social and emotionally-affected behaviors, have been reported. Here, we present evidence that VPMNN collaterals also project to the amygdaloid complex, and establish synaptic connections with neurons in central amygdala (CeA). The density of AVP innervation in amygdala was substantially increased in adult rats that had experienced neonatal maternal separation (MS), consistent with our previous observations that MS enhances VPMNN number in the paraventricular (PVN) and supraoptic (SON) nuclei of the hypothalamus. In the CeA, V1a AVP receptor mRNA was only observed in GABAergic neurons, demonstrated by complete co-localization of V1a transcripts in neurons expressing Gad1 and Gad2 transcripts in CeA using the RNAscope method. V1b and V2 receptor mRNAs were not detected, using the same method. Water-deprivation (WD) for 24 h, which increased the metabolic activity of VPMNNs, also increased anxiety-like behavior measured using the elevated plus maze (EPM) test, and this effect was mimicked by bilateral microinfusion of AVP into the CeA. Anxious behavior induced by either WD or AVP infusion was reversed by CeA infusion of V1a antagonist. VPMNNs are thus a newly discovered source of CeA inhibitory circuit modulation, through which both early-life and adult stress coping signals are conveyed from the hypothalamus to the amygdala.

## Introduction

Arginine-vasopressin (AVP)-containing magnocellular neurosecretory neurons (VPMNNs) have a critical role in the control of water-electrolyte balance via AVP release from the neurohypophysis, and also influence fundamental behaviors important for survival, presumably at the level of the brain itself (Greving, 1923, 1928; Harris, 1948; de Wied, 1979; Koob et al., 1981; Landgraf and Neumann, 2004). However, despite the profound behavioral and physiological effects of central AVP (Mühlethaler et al., 1982; Landgraf and Neumann, 2004), and early attempts to characterize the central projections of the VPMNN (Buijs, 1978, 1980; DeVries et al., 1985), we remain relatively uninformed about the central organization of the underlying AVP neuronal connections within the central nervous system (CNS): i.e., physiological circuit interactions, synaptic innervation patterns, and the identity of post-synaptic neuronal targets.

One area of the brain in which AVP likely exerts profound behavioral effects is the amygdala. The amygdala is a complex structure involved in anxiety and fear processing (Davis and Whalen, 2001; LeDoux, 2007). In particular, the central amygdala (CeA) is thought to be critical for emotional processing of environmental input, especially in regard to plasticity of behavioral responses to stress (Penzo et al., 2015). A potential target for AVP input to CeA are its numerous GABAergic neurons (Sun and Cassell, 1993; Swanson and Petrovich, 1998), which are important for expression of anxiogenic responses (Sun et al., 1994; Pará et al., 2004; Huber et al., 2005).

There are well-documented AVP projections to amygdala (Buijs, 1978, 1980; Buijs and Swaab, 1979; Buijs et al., 1983; Caffé and van Leeuwen, 1983; Rood and De Vries, 2011; Zhang and Hernández, 2013), and AVP elevation in CeA is correlated with maternal aggression and other stress-related behaviors (Landgraf and Neumann, 2004). However, the origin, distribution, and co-localization with classical neurotransmitters of these fibers, and behavioral consequences of activation of AVP release from these fibers, especially during development, have received little attention. In particular, it has only been demonstrated recently that dynamic regulation of VPMNNs of the hypothalamic paraventricular (PVN) and supraoptic (SON) nuclei may convey stressor response signals to non-neurohypophyseal limbic regions (Hernández and Zhang, 2012; Zhang et al., 2012; Zhang and Hernández, 2013; Hernández et al., 2015). Thus, we have now begun to characterize the VP-MNN projections to the amygdala, and investigate their involvement in developmentally-related and adult stress responses.

Perinatal stress in rodents, such as maternal separation (MS), fragmented maternal care, and maternal endocrine dysfunction, has been used to examine the causal relationship between early life stress and later stress overreactivity and susceptibility to affective disorders (Zhang et al., 2010, 2012; Lukas et al., 2011). AVP is among several neurotransmitter systems reported to be altered as a consequence of early life stress (Veenema et al., 2006, 2007; Lukas et al., 2011), and therefore is among the neurotransmitter candidates for mediating the reported later-life behavioral hyperreactivity associated with early life stress. We have reported that the AVP system is activated as a consequence of stress during the early life period, with increased VPMNN PVN and SON cell number, enlargement of Herring body densities, and increased number of reciprocal synaptic connections between vasopressinergic and corticotropin releasing factor (CRF) containing neurons. This neuronal plasticity is associated with increased sensitivity to acute stressors, and anxiogenic conditions in adulthood (Zhang et al., 2010, 2012).

In the present study, we have combined immunohistochemistry with antero- and retrograde tracing, electron microscopy and anatomical analysis, to show that AVP innervation of the amygdaloid complex has at least dual origins: thin fibers likely arising from a small population of neurons localized in the bed nucleus of stria terminalis intra-amygdaloid division (STIA), likely to establish Gray type II synapses; and thick fibers originating from hypothalamic PVN and SON, positive for vGluT2 and establishing Gray type I synapses. Semiquantitative anatomical analysis in combination with AVP-IHC showed that the medial amygdala (MeA) was the region with the densest AVP innervation followed by the rostral part of the CeA, where V1a receptor-positive GABAergic neurons were located. MS produced a substantial increase in AVP immunopositive fibers, especially in the central and medial amygdala. We also found that in the CeA the V1a receptors were only expressed in GABAergic neurons. Finally, physiological and pharmacological manipulations of vasopressinergic neurotransmission from PVN to CeA were used to show the functional importance of this projection to the modulation of anxious behavior in the rat. In this regard, we examined the effects on vasopressinergic projections from the hypothalamic VPMNNs to the amygdala, of at least one physiological condition, water deprivation (WD), for which neurohypophyseal AVP (hormone) release is a known homeostatic response, in order to determine if there is a corresponding allostatic response to WD in the amygdala. Here, we report that this is indeed the case, and discuss its implications for dual vasopressinergic homeostatic regulation via the neurohypophysis, and allostatic regulation via VPMNN projections to extrahypothalamic brain, including the amygdala.

## Materials and Methods

### Animals

Adult male Wistar rats of 280 ± 20 g were obtained from the local animal facility. Animals were housed three per cage under controlled temperature and illumination (12 h/12 h, inverted to the natural light/dark cycle) with water and food *ad libitum*. All animal procedures were approved by the Comision de investigacion y Etica de la Facultad de Medicina, Universidad Nacional Autonoma de Mexico (approval number: CIEFM-086-2013).

### Maternal Separation Protocol

The MS (3 h daily, MS 3 h) procedure is described in detail elsewhere (Zhang et al., 2012). Briefly, female and male adult rats were mated for 2 days. During the last week of gestation, female rats were single-housed in standard rat Plexiglas cages and maintained under standard laboratory conditions. On the day after parturition, postnatal day (PND) 2, each litter was culled to 7–8 pups, in which 5–6 were males. During the period from PND 2–PND 16, the pups were separated daily from their dams, and placed into an incubator at 29°C ± 1°C, between 09:00 h and 12:00 h. After this period rats were returned to their home cages. After ending the MS protocol, animals were left undisturbed until the weaning at PND 28, when male and female rats were separated. Animals were then left undisturbed until PND 90 when the elevated plus maze (EPM) was performed during their activity period. Animal facility reared (AFR) rats were treated in the same conditions as described above except that these animals were left undisturbed in their cages during the period when MS 3 h rats were separated from their dam. Bedding was changed twice a week for both groups, with minimum disturbance to the rats.

### Immunohistochemistry

Rats were deeply anesthetized with an overdose of sodium pentobarbital (63 mg/kg, Sedalpharma, México) and perfused transaortically with 0.9% saline followed by cold fixative containing 4% of paraformaldehyde in 0.1 M sodium phosphate buffer (PB, pH 7.4) plus 15% v/v saturated picric acid for 15 min (for the immunoreaction using monoclonal antibody against GABA, 0.1% glutaraldehyde was also added to the fixative solution). Brains were immediately removed, blocked, and then thoroughly rinsed with PB. Brains were sectioned soon after perfusion using a Leica VT 1000S vibratome. Freshly-cut freely-floating sections were blocked with 20% normal donkey serum (NDS) in Tris-buffered (0.05 M, pH 7.4) saline (0.9%) plus 0.3% of Triton X-100 (TBST) for 1 h at room temperature and incubated with the primary antibodies listed in Table 1 (for antibody specificity see “Supplementary Material Table S1”). For light microscopical immunohistochemistry, Vectastain Elite ABC Kit was used (Vector Labs, Burlingame, CA, USA) followed by DAB-peroxidase reaction, while for immunofluorescence reactions, sections were incubated with the corresponding fluorochrome-conjugated secondary antibodies. For immunoreaction visualization, a Nikon Eclipse 50i light microscope and a Leica TCS-SP5 confocal microscope were used.

**Table 1:** **Primary antibodies used in this study**.

Molecule	Host	Dilution	Source	Source code
[Arg8]-vasopressin	Rabbit	1:5000	Peninsula-Bachem Americas, Inc., CA, USA (www.bachem.com)	T-4563
[Arg8]-vasopressin	Rabbit	1:2000	Prof. R.M. Buijs, Instituto de Investigaciones Biomédicas, Universidad Nacional Autónoma de México, UNAM	–
c-Fos	Rabbit	1:2000	Santa Cruz Biotechnology, Dallas, TX, USA (www.scbt.com)	SC-52
Vesicular glutamate transporter 2	Guinea pig	1:1000	Frontier Institute Co., Ltd., Hokkaido, Japan (www.frontier-institute.com)	GP-AF240-1
Gamma-aminobutyric Acid (GABA)	Mouse	1:1000	Sigma-Aldrich Corporation, St. Louis, MO, USA (www.sigmaaldrich.com)	A031
Vasopressin V1A Receptor	Rabbit	1:2000	Alomone Labs., Jerusalem, Israel (www.alomone.com/)	AVR-010

### Anatomical Assessment of AVP Innervation Density in Amygdala Subfields and Comparison Between MS and AFR Rats

Observations were made under light microscopy. Anatomical nomenclature, especially on amygdala subfields, and regional delineation were according to Paxinos and Watson (2006). To evaluate the densities of vasopressin-containing axon projections in amygdala, observations were made under a light microscope (Nikon Eclise, 50i, with 40× objective) and tracing was performed with the help of a drawing tube attached to the microscope (amplification at 10.6×). A square equivalent to 0.04 mm^2^ of the brain tissue section was placed under the drawing tube projecting to the visual field coinciding with the anatomical regions of interests. AVPir+ fibers inside the projected square were manually traced. Calculation of fiber length in each square was performed using ImageJ (open image > make binary > measure). The value of the area in the results window corresponds to the number of pixels in the image. Since the process of skeletonization converts every trace into a one pixel thick trace, the number of pixels equals the summed length of the traces in pixels. The real length is converted to millimetre taking into account the correspondence between pixels and a known scale value. AVPir+ fiber length from postero-dorsal medial amygdala (MePD) from coronal section (Bregma. −2.7 mm) was 3618 mm and was assigned as 100%. Fiber lengths falling in the range of 100%–76% were assigned as ++++; 75–51% as +++; 50–26%: as ++ and 25–1% +.

### Neuronal Activation Through Fos-Expression Assessment

The pattern of amygdala neuronal activation after the EPM test was evaluated in four animals for each of the cannulated rat groups, i.e., those infused with NaCl 0.9%, with AVP 1 ng, or with AVP 1 ng + V1a antagonist 10 ng. The number of Fos+ nuclei in an area of 0.2 mm^2^ was counted in the central (CeA), basolateral (BLA) and MeA, around Bregma −2.12 mm. A two way analysis of variance (ANOVA) was used to evaluate if the differences between the NaCl, AVP 1 ng and AVP 1 ng + V1a antagonist groups were statistically significant.

### Immunohistochemistry for Transmission Electron Microscopy (TEM)

Immuno-electron microscopy procedures were performed as reported previously (Zhang and Hernández, 2013). Briefly, two male rats were perfused with NaCl 0.9%, and fixed with ice-cold 4% paraformaldehyde (Sigma P6148) + 15% v/v saturated picric acid solution (Fermont) + 0.05% glutaraldehyde (Sigma, G5882). Sections 70 μm-thick containing CeA were processed with conventional IHC for TEM (Zhang and Hernández, 2013). Sections were then osmificated in 1% OsO_4_ for 1 h and dehydrated in a graded series of ethanol and embedded in synthetic resin (Epon). Ultrathin sections were then cut, counterstained with lead citrate (Electron Microscopy) and examined with a Philips CM 100 transmission electron microscope and photographed with a digital micrograph 3.4 camera (Gatan Inc., Pleasanton, CA, USA).

### Fluoro-Gold Tracing Experiments

Anesthesia was induced and maintained with xylazine (Procin, Mexico; 20 mg/ml) and ketamine (Inoketam, Virbac, Mexico; 100 mg/ml) mixed in a 1:1 volume ratio and administered intramuscularly with a dose of 1 ml/kg body weight. Rats were fixed in a stereotaxic apparatus and were injected in the CeA (Bregma −2.12 mm, medio-lateral 4.2 mm, dorsoventral 7.7 mm (Paxinos and Watson, 2006)) with the retrograde tracer Fluoro-Gold (FG, Fluorochrome, LLC, Denver, CO, USA), dissolved 1% in 0.2 M sodium cacodylate buffer (pH 7.5). The FG was delivered iontophoretically using an iontophoresis pump (Value Kation Sci VAB-500) through a stereotaxically positioned glass micropipette with an inner tip diameter of approximately 40 μm, by applying current pulses of 0.1 μA, at 0.2 Hz, with a 50% duty cycle, for 20 min. The micropipette was left in place for an additional 10 min to prevent backflow of the tracer up the injection track after each injection. After completing the surgery, rats received 0.4 mg/kg *i.p*. Ketorolac (Apotex, Mexico) and 50 mg/kg *i.p* ceftriaxone (Kendric, Mexico) as analgesic/anti-inflammatory and antibiotic agents, respectively. Three to four weeks after the FG injections, the rats were perfused as previously described (Zhang and Hernández, 2013). Coronal and sagittal sections of 70 μm were obtained, and AVP IHC was performed to evaluate if the SON and PVN AVP+ neurons were labeled with FG. Observations were made under light (Nikon ECLIPSE 50i with B-2A long-pass emission filter) and confocal microscopy (Leica TCS-SP5).

### ISH Assays With Radio-Probes

Rats were deeply anesthetized with sodium pentobarbital (Sedalpharma, México, 63 mg/kg b.w., i.p.) and perfused via ascending aorta with 0.9% saline followed by cold fixative containing 4% paraformaldehyde in 0.1 M PB, pH 7.4. Brains were post-fixed with 1% paraformaldehyde in PB and kept at 4°C until use. All solutions used had been diethyl pyrocarbonate (DEPC)-treated (0.1% v/v with gentle stirring for at least 4 h at room temperature) to inactivate any residual RNase, and then autoclaved to inactivate the traces of DEPC.

Two days before the start of sectioning, the brains were moved to 18% sucrose in RNase free PB + NaN3. Another change was done 1 day before the sectioning and a third change with a fresh sucrose solution was done 1 h before the sectioning. Serial coronal sections between Bregma −0.36 mm and Bregma −3.00 mm of 12 μm of thickness were made using a Leica CM1950 cryostat (Leica Microsystem, Wetzlar, 35578, Germany). Sections were collected on Leica glass insert and then transferred to a 24-well tissue culture plate with PB.

*In situ* hybridization (ISH) was performed in one out of six coronal sections as previously described (Morales and Bloom, 1997) using ^35^S- and ^33^P-UTP-labeled ribonucleotide probes. The pT7T3D-PacI plasmid (accession number: AI072073, clone ID: 1786383 Thermo Scientific) containing rat vasopressin receptor V1a cDNA (Morel et al., 1992) was linearized with EcoRI and then transcribed *in vitro* with T3 RNA polymerase to yield antisense complementary RNA probe. The construct was verified by sequencing. The radioactivity was adjusted to 10^7^ cpm per ml hybridization buffer. Sections were mounted on coated slides and air-dried. Slides were exposed to autoradiography film and analyzed on a phosphorimager (Fuji BAS5000, Tokyo, Japan).

### RNAscope ISH Assays

Two rats were deeply anesthetized and perfused via the ascending aorta with saline. Whole brain tissues were removed and rapidly frozen on Dry Ice. The fresh-frozen tissue sections (thickness: 20 μm) were mounted on positively charged microscopic glass slides (Fisher Scientific, Pittsburgh, PA, USA). The Gad1, Gad2 and V1a specific RNA probes (Rn-Gad1, 316401-C1; Rn-Gad2, 435801-C2; Rn-Avp1a, 402531-C3) were designed and provided by Advanced Cell Diagnostics (Hayward, CA, USA). All staining steps were performed following RNAscope protocols. Stained slides were cover-slipped with fluorescent mounting medium (ProLong Gold Antifade Reagent P36930; Life Technologies, Carlsbad, CA, USA) and examined with a confocal microscope (Leica TCS-SP5).

### Intra-Amygdaloid Microinjection

For the implantation of permanent guide cannulas into the amygdala, rats were anesthetized with ketamine hydrochloride (170 mg/kg, i.p.) and placed in a stereotaxic frame (Kopf Instruments, Tujunga, CA, USA) with the incisor bar set at −3.3 mm. Body temperature was maintained at 37°C using a CMA/150 temperature controller (CMA/Microdialysis, Stockholm, Sweden). Bilateral stainless steel cannulae of 0.46 mm outer diameter (C315G, Plastics One, Roanoke, VA, USA) were aimed to CeA (Bregma −1.7 mm, mediolateral ± 4.2 mm, dorsoventral −7.7 mm from skull surface, according to the atlas of Paxinos and Watson (2006). Guide cannulas were fixed with stainless-steel screws and dental acrylic cement (Laboratorios Arias, Mexico City, Mexico) and sealed with dummy cannulae (C315DC, Plastics One). Benzylpenicillin benzathine (Estrepto benzetacil V-Fortificado (Fort Dodge Animal Health Laboratories, Mexico City, Mexico)) was given to prevent infection. The animals were housed in individual cages post-surgery. A total of 30 rats were employed for microinjection experiments.

Beginning on the fourth day post-surgery, rats were handled once a day for 5 min for three consecutive days, and behavioral tests were performed the next day (seventh day post-surgery).

On the day of the experiment, saline alone (250 nl per side), AVP (Sigma V9879, 1 ng in 250 nl per side of NaCl 0.9%, (Appenrodt and Schwarzberg, 2000) or V1a antagonist (d(CH_2_)_5_ 1, Tyr(Me) 2, Arg^8^)-Vasopressin trifluoroacetate salt, Manning compound; 10 ng in 250 nl per side), depending the experimental groups, were injected bilaterally via an injection cannula (0.20 mm outer diameter, C315I, Plastics One), which protruded 1 mm beyond the end of the guide cannula, over a period of 5 min (Pérez de la Mora et al., 2006), using two CMA/Microdialysis pumps (CMA/Microdialysis, Stockholm, Sweden). The cannulae were kept in place for 1 min after the injection to prevent backflow and to allow for diffusion. Behavioral observations were started 15 min after microinjections.

### Elevated Plus Maze

The EPM was made locally (for details see Zhang et al., 2010, 2012). The test was performed during the early activity period under dim red light and video recorded. Briefly, testing is performed by placing the rat in the center of the maze facing towards an open arm, and free exploratory activity is observed for 5 min. An entry into an open arm was counted when the four paws of the rat were placed within that arm. The time spent in the open arms, as percentage of total time (300 s), was analyzed off-line. For each treatment, the person administering the drug was aware of the animal’s status (i.e., control-reared or MS-reared). The persons who performed behavioral assessments were unaware of either the rearing or treatment status of each assessed animal.

Two sets of experiments (“Experiment 1” and “Experiment 2”) were performed. Experiment 1: MS (*n* = 10) and AFR (*n* = 10) rats underwent a 24 h water-deprivation period (WD 24 h) previous to the EPM test. The rationale of this set of experiments was that WD 24 h potently up-regulates the metabolic activity of the hypothalamic VPMNNs but with only a modest increase in plasma osmolarity (Dunn et al., 1973; Zhang et al., 2012). Moreover, we have previously reported that MS rats possess a potentiated VPMNN system in adulthood, with enhanced anxiety state as measured by the Vogel conflict test, after WD 24 h (Zhang et al., 2012). Experiment 2: three experimental groups of AFR rats were assessed under EPM paradigm. Group A: microinjection with saline; group B, microinjection with AVP and group C, microinjection with AVP and the V1a specific antagonist.

### Statistical Analysis

Quantitative results were expressed as mean ± SEM. Groups were tested for normality with a D’Agostino and Pearson test. Differences between means were evaluated by one-way ANOVA followed by the Bonferroni test. All computations were done using Prism (GraphPad Software, Version 6, San Diego, CA, USA). Differences in all cases were considered statistically significant when *P* < 0.05.

## Results

### Anatomical Features of Arginine-Vasopressin Containing Somata and Fibers in the Amygdaloidal Complex as Revealed by Immune-Reaction (AVPir)

**Figure 1:**
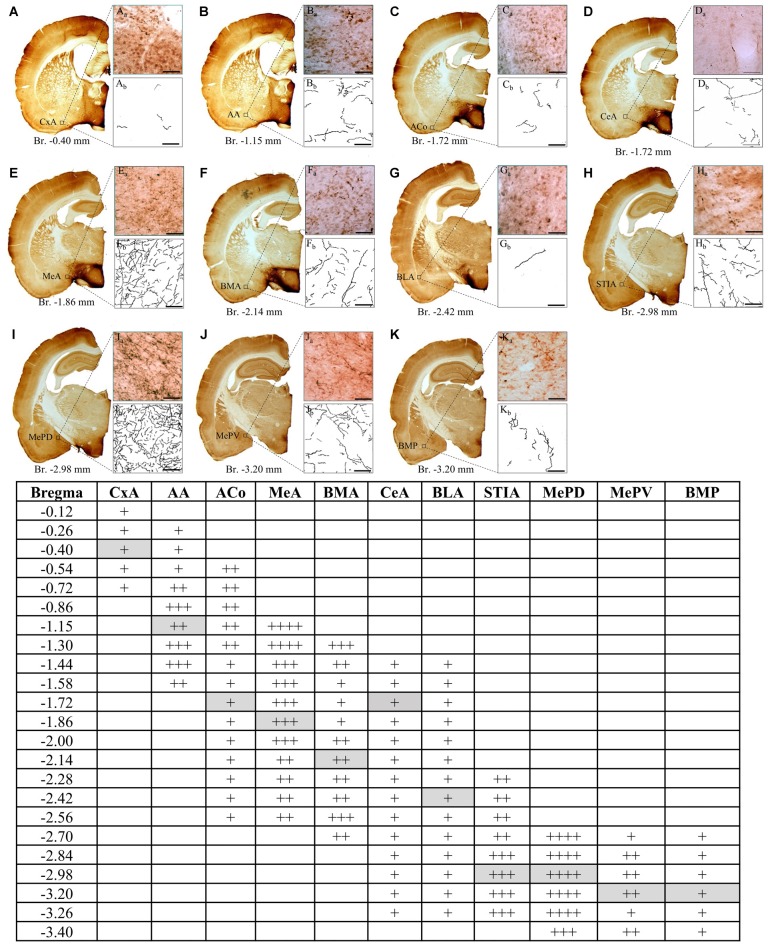
**Distribution of vasopressin-immunostained fibers through the rostrocaudal extent of amygdala in the rat.** Low **(A–K)** and high power **(A_a_–K_a_)** photomicrographs and tracings of arginine-vasopressin (AVP) labeled fibers **(A_b_–K_b_)** in coronal sections, illustrating the heterogeneous distribution in the density of AVP fibers among different amygdalar regions. The highest density was found in the MePD region at Bregma level −2.70 mm (panel **I**, and table). Each of the panels from **(A–K)** corresponds to a representative example at the bregma level indicated in the table gray boxes. For the semiquantitative analysis in the table, the summed length (calculated with ImageJ) of fibers in a 0.04 mm^2^ area, inside this highest density region was assigned as 100%, regions with summed lengths between 76–100% were assigned as ++++; between 51–75% : +++; between 26–50%: ++ and between 1–25%: +. Anatomical maps and nuclei nomenclature are from the rat atlas of Swanson (2003). CxA, cortico-amygdalar transition zone; AA, anterior amygdala area; Aco, anterior-cortical amygdaloid nucleus; MeA, medial anterior amygdaloid nucleus; BMA, basomedial anterior amygdaloid nucleus; CeA, central nucleus of amygdala; BLA, basolateral amygdala; STIA, stria terminalis intra-amygdaloid division; MePD, medial postero-dorsal amygdaloid nucleus; MePV, medial postero-ventral amygdaloid nucleus; BMP, basomedial posterior amygdaloid nucleus. Scale bars in **(A_a_–K_a_, A_b_–K_b_**) 50 μm, Bregma levels are indicated below each low power photomicrograph. Squared region indicates the 200 μm area used for calculation of summed length for each amygdala region.

We used an AVPir-optimized protocol previously designed by us (Zhang and Hernández, 2013) to reveal the AVP-containing fibers and somata distribution. We observed, as shown in Figure 1, a heterogeneous distribution of fibers in the whole amygdaloid complex, with the medial MePD, the MeA (rostral to MePD), the bed nucleus of STIA and the anterior amygdala (AA, rostral to CeA) being the most densely innervated regions.

**Figure 2:**
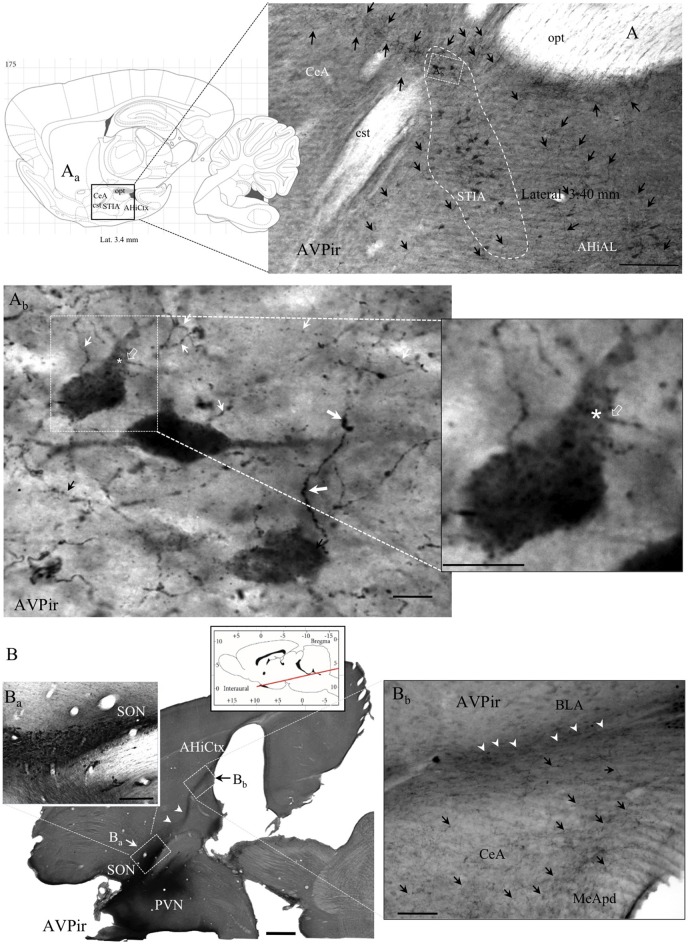
**Anatomo-immunohistochemical features of vasopressin immuno-reactive (AVPir) somata and fibers in the amygdaloidal complex suggest that AVP modulates distinct neuronal circuits. (A)** Photomicrograph of AVPir in a sagittal section of rat amygdala at lateral 3.40 mm (**A_a_**, inset: sagittal view of amygdaloid complex, squared region, from the Rat Brain Atlas). Note that the AVP immunopositive fibers (indicated by black arrows) are heterogeneously distributed in the whole amygdala complex and numerous AVP+ cells were inside the bed nucleus of *stria terminalis*, intra-amygdaloid division (STIA; circumscribed with white dashed line). **(A_b_**) Amplification of a region inside the STIA. Note that there are two types of AVPir+ fibers, regarding their thickness and spatial frequency of varicosities: the type I fibers have thick diameter and frequent varicosities (thick white arrows); the type II fibers are thin and had less frequent varicosities (thin white arrows). Both types are intermingled in the same region. The asterisk indicates the site where one thin-type axon (the axon initial segment was indicated with a hollow arrow, white in both **(A_b_**) and its inset) originated (asterisk) from one proximal dendrite of the respective neuron. **(B)** A semi-horizontal section of rat brain showing the vicinity of the hypothalamic AVP+ supraoptic (SON) and paraventricular (PVN) nuclei and the massive projection of AVPir fibers toward amygdala and amygdala-hippocampal cortex (AHiCtx; white arrowheads in **B_a_,B_b_**). The red line of the atlas inset symbolizes the level and the inclination of the section plan. **(B_a_**) Amplification of the corresponding squared region showing the SON is seen from the semi-horizontal view. **(B_b_**) Amplification of the corresponding squared region of amygdaloid complex. Note the heterogeneous presence of the AVPir fibers in the amygdaloid subdivisions. MeApd, medial amygdala postero-dorsal; CeA, central amygdala; BLA, basolateral amygdala. Scale bars: **(A)** 500 μm; **(A_b_**) and Inset: 20 μm; **(B)** 500 μm; **(B_a_**) 100 μm; **(B_b_**) 100 μm.

AVPir somata were observed in the STIA and surrounding regions (Figure 2A dashed line circumscribed region). These cells were sparsely distributed and had pale AVPir labeling. AVPir + fibers all over amygdala showed either a strong-dark labeling or a weak-pale labeling pattern. Two kinds of fibers were observed. The first kind of fiber was varicose, and was the most frequently observed fiber type (Figure 2A_b_). The second kind was rarer, and less varicose. We will refer to these as Type I and Type II fibers, respectively, in accordance with the previous nomenclature that we used for hippocampal AVP innervation (Zhang and Hernández, 2013). Some type II axon proximal segments could be found leaving AVPir somata (Figure 2A_b_ inset). Using semi-horizontal sections, we could clearly appreciate the limits between the hypothalamic PVN, SON and amygdala. Figures 2Bs show the massive projection and the heterogeneous distribution patterns of AVPir fibers towards amygdala/amygdalo-hippocampal cortex.

### Effect of Maternal Separation on Density of AVP Innervation in Amygdala

**Figure 3:**
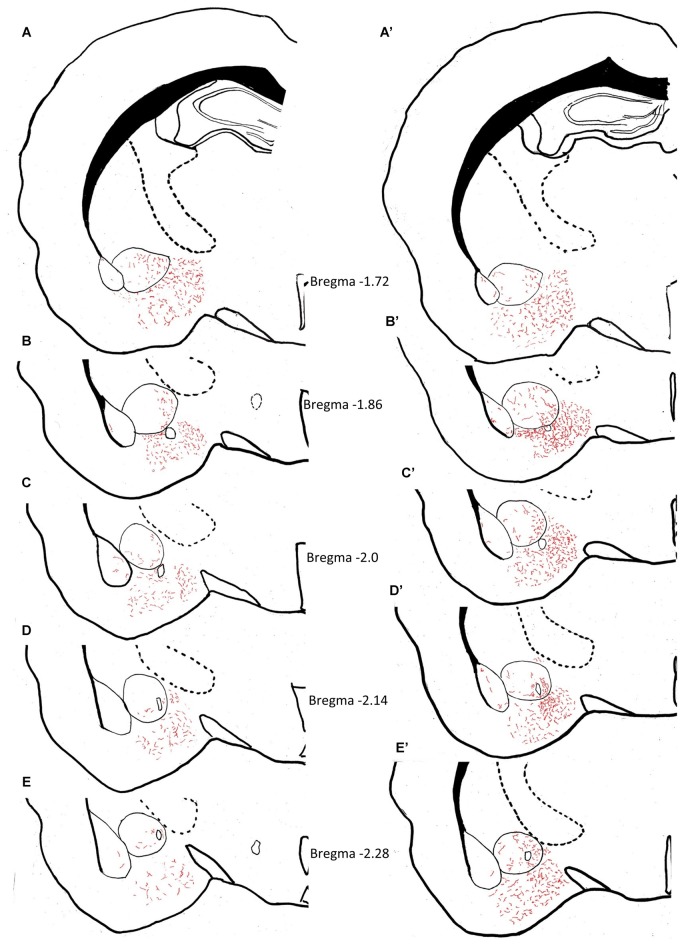

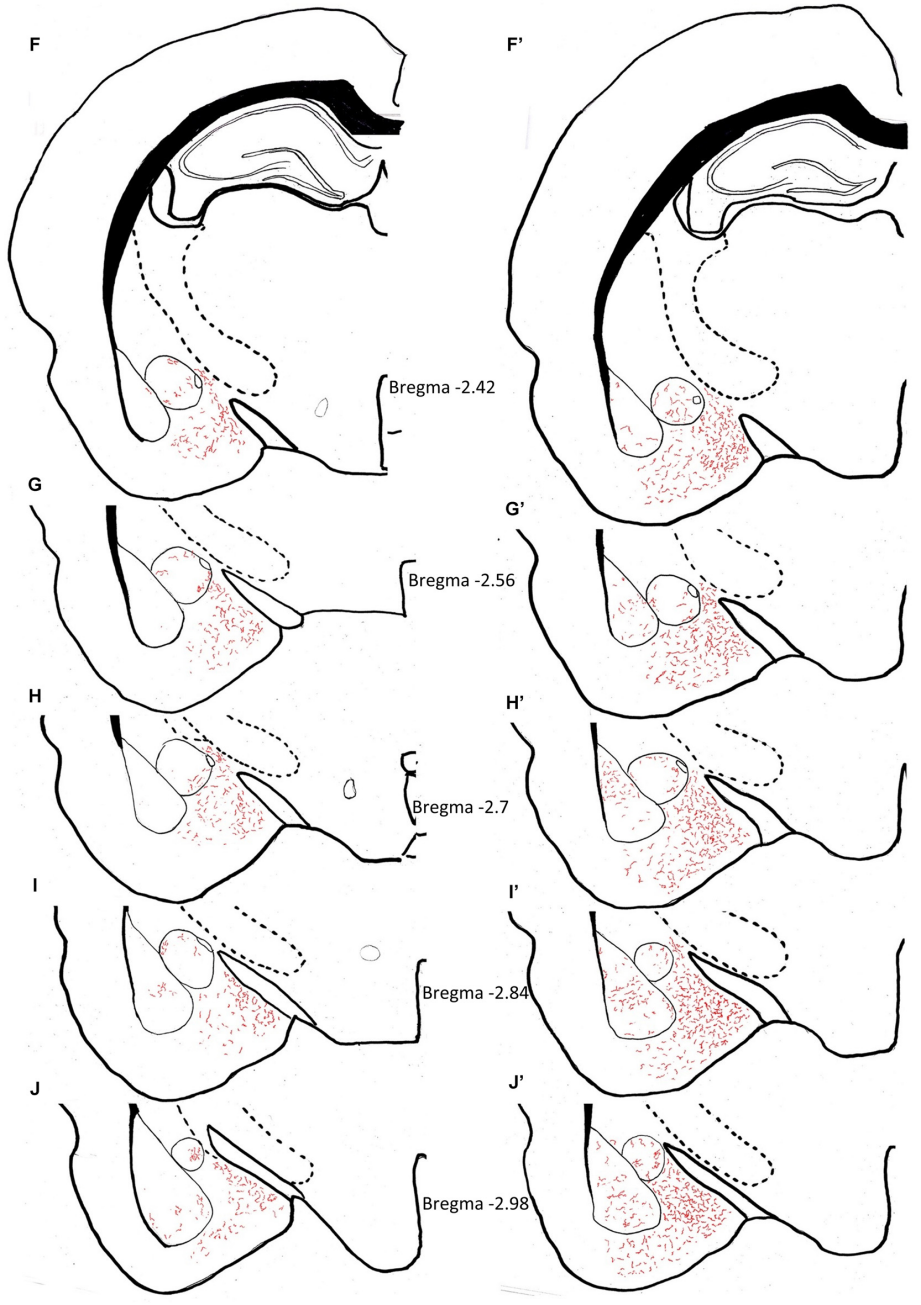
**Anatomical charting of AVPir+ fiber distribution in amygdala in both control (A–J)** and maternal separation (MS) male adult rat (MS, **A’**–**J’**). Chartings of coronal sections at 10 rostro-caudal levels with line drawings referenced with microscopic observation, representing AVP fiber distribution through the entire amygdaloidal complex. Note the remarkable increase in AVP innervation densities in all regions of the amygdala as a function of MS.

The density of AVP innervation in amygdala was substantially increased in adult rats who had experienced neonatal MS, consistent with our previous observations that MS enhances VPMNN number in the PVN and SON nuclei of the hypothalamus. Using anatomical analysis with the help of light microscope and a drawing tube, we produced a coordinate-detailed anatomical chartings of AVP innervation in adult male rat amygdala (Figures 3A–J). The density of AVP innervation in amygdala was substantially increased in adult rats that had experienced neonatal MS (Figures 3A’–J’ and Table 2), consistent with our previous observations that MS enhances VPMNN number in the PVN of the hypothalamus.

**Table 2:** **Vasopressin fiber density distribution in animal facility reared (AFR) and MS rats**.

Control				Maternal separation			
Bregma (mm)	BLA	CeA	MeA	Bregma (mm)	BLA	CeA	MeA
−1.72	+	++	+++	−1.72	+	++	++++
−1.86	+	++	+++	−1.86	+	++	++++
−2	+	++	+++	−2	+	++	++++
−2.14	−	+	+++	−2.14	+	++	++++
−2.28	+	+	+++	−2.28	+	++	++++
−2.42	+	+	+++	−2.42	+	++	++++
−2.56	−	+	+++	−2.56	++	+	++++
−2.7	−	+	+++	−2.7	++	+	++++
−2.84	+	+	+++	−2.84	++	++	++++
−2.98	+	++	+++	−2.98	++	+	++++

*Tracing was performed under camera lucida and calculation of axonal length in a determined region was calculated using ImageJ. Axon length value from Medial Amygdala (MeA) at Bregma level −1.86 mm in maternal separation (MS), was asigned to 100% (++++) = MAX value. For each region, ++++, +++, ++ and + semi-quantitative values were assigned for fiber length values 76–100%, 51–75%, 26–50% and 1–25% of the MAX value*.

### Immuno-Electron Microscopical Evidence of Synaptic Innervation by Thick and Thin AVP-Containing Fibers onto Central Amygdala (CeA) Neurons, Establishing either Gray Type I (Asymmetric; Thick) or Gray Type II (Symmetric; Thin) Synapses

**Figure 4:**
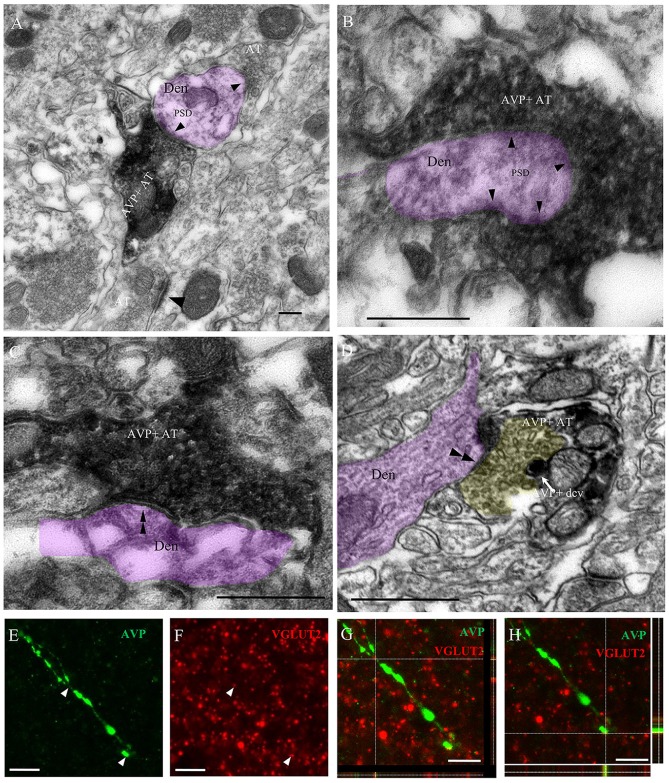
**Immuno-electron microscopical evidence of synaptic innervation by thick and thin AVP-containing fibers onto central amygdala (CeA) neurons, establishing either Gray type I (asymmetric; thick) or Gray type II (symmetric; thin) synapses.** Examples of thick axon terminals (AT) **(A,B)** making Gray type I synapses (the presence of postsynaptic density, PSD, are indicated by single arrowheads) and thin AT **(C,D)** making Gray type II synapses (the absence of PSD are indicated by double arrowhead) onto CeA neurons’ dendrites (Den, pink shaded). Note that the yellow shaded presynaptic active zone of an axon terminal of a thin fiber contained pleomorphic small clear vesicles, which is a remarkable feature of a GABAergic AT; it also contained AVPir+ dense core vesicle (dcv). Thick AVPir fibers in the CeA contain vesicular glutamate transporter (VGLUT2)-positive varicosities. Confocal microscopy analysis of CeA showed thick varicose fibers immunopositive for AVP **(E)** and multiple VGLUT2-positive terminals **(F)** with co-expression of VGLUT2 and AVP in a representative thick fiber **(G,H)**. For **(G,H)**, the *x–y* (center), *x–z* (box below) and *y–z* (right box) images are shown. Scale bars: **(A–D)** 500 nm; **(E–H)** 10 μm.

Ultrastructural analysis of the AVP innervation showed that thin and thick AVP immunopositive fibers, with dense core vesicles (dcv, AVPir+), made synapses onto dendrites in the CeA. The axon terminals (AT) of the thin fibers presented pleomorphic clear vesicles characteristic of GABAergic AT and preferentially made presumptive inhibitory (GABAergic) symmetric “Gray type II” synapses, while the thick fibers made presumptive excitatory (glutamatergic) asymmetric “Gray type I” synapses (Figures 4A–D). To further confirm the excitatory nature of the thick fibers in amygdala, double immunohistochemistry against AVP and the vesicular glutamate transporter (VGLUT2) was performed. Confocal microscopical analysis in the CeA showed that thick varicose axonal fibers containing AVP co-expressed VGLUT2 (Figures 4E–H).

### Anterograde and Retrograde Tracing Revealed that PVN and SON Magnocellular Vasopressinergic Neurons Innervate the Central Amygdala

**Figure 5:**
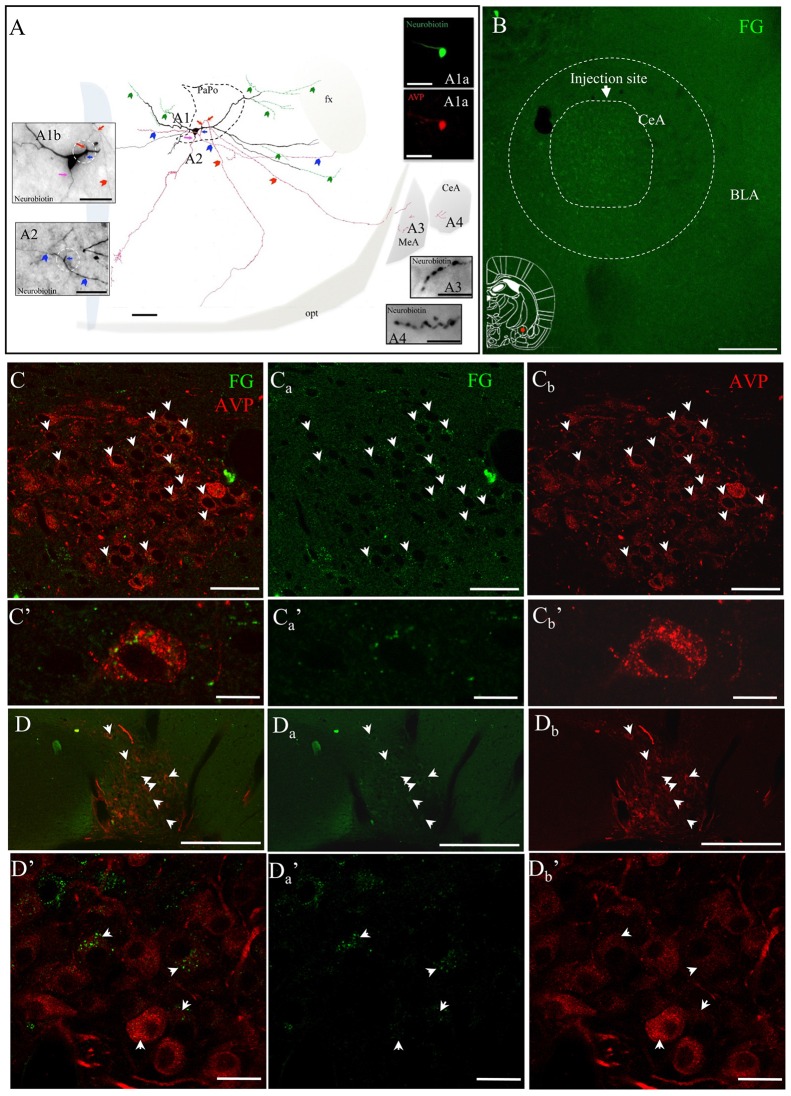
**AVP-containing magnocellular neurons innervate the CeA. (A)** Camera lucida reconstruction of the coronal projection and corresponding photomicrographs (insets) showing the dendritic and axonal patterns of an anterogradely labeled AVP-containing magnocellular neurosecretory neuron sending axon collaterals to the medial and CeA. This panel was modified from Hernández et al. (2015), see the original publication for method descriptions. Red, blue and pink arrows indicate the origins of three parent-axons. Red and blue arrowheads indicate the subsequent axonal branches. The green arrowheads indicate the beaded processes originated from the dendritic processes, some of them entering the fornix (green arrowheads). Such processes were described in an early study and were considered as axonal processes (Sofroniew and Glasmann, 1981). **(A1a)** AVP-containing nature was ascertained by AVP immunoreaction; **(A1b)** neurobiotin-peroxidase-AB complex histochemical reaction showing the morphology of the filled AVPir+ neuron’s soma and its three parental axons emitted from proximal dendrites (pink, red and blue arrows indicate the sites where the axons were originated). Note that two of those axons were emitted from the same proximal dendritic point (red and blue arrows but coursed to opposite directions). **(A2)** Photomicrograph of the section contiguous caudally to the section of soma **(A1b)** showing the branching of the axon collaterals (colors are aimed to encode each group of collaterals originated from the same parental main axon). **(A3,A4)** show axonal processes found in amygdala. Scale bars: 100 μm for **(A)** 50 μm for all photomicrographs. MeA, medial amygdala; CeA, central amygdala; opt, optical tract; fx, fornix. Panel **(B)** Fluorogold (FG; retrograde neural tracer) was iontophoretically injected into CeA. **(Cs)** panels correspond to the retrograde labeling images of the PVN with FG, AVPir and overlapping of the two channels respectively. **(C’s)** show an example of a magnocellular AVPir+ neuron in high magnification. Note that the green dots, which correspond to FG, are peri-nuclear which likely are intra-lisosomal. **(D,D’)** are the corresponding images for supraoptic nuclear labeling. Scale bar: 50 μm.

The presence of two AVP+ fiber populations, with distinct diameters and synaptic profiles, suggested that the AVP innervation in amygdala could have different origins. To assess this, fluorogold (FG) was stereotaxically injected in the CeA. Three to four weeks following the FG injection, rats were perfused, brains were sectioned, immunohistochemistry against vasopressin was performed, and areas of interest were evaluated under a confocal microscope. Cases in which the injection area was sufficiently focused (<500 μm in diameter), and localization of the injection site to CeA was achieved, were evaluated. A number of AVP+ neurons in the PVN and SON showed clear FG labeling, mainly accumulated in the perikarya of lysosome-like granules (Figures 5B–D), which is a prominent characteristic reported for successful long-term FG labeling (Schmued and Fallon, 1986; Wessendorf, 1991; Persson and Havton, 2009).

These results confirmed previous observations of our group (Hernández et al., 2015), in which some axons of an *in vivo* juxtacellularly labeled AVP+ magnocellular neuron from hypothalamic PVN nucleus, reconstructed under a camera lucida, were seen reaching the CeA and MeA (Figure 5A).

### An Increase in Anxious Behavior Observed After Water Deprivation of 24 h (WD 24 h) and Further Significantly Increased in MS Subjects

**Figure 6:**
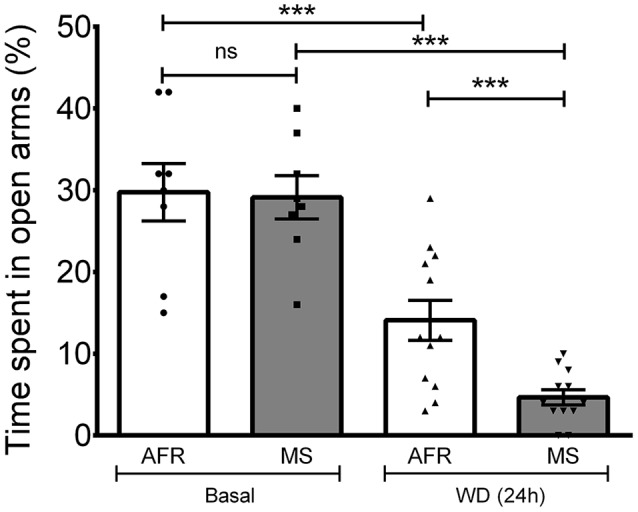
**Elevated plus maze (EPM) performance after AVP magnocellular system upregulation induced by 24-h water deprivation (WD, 24 h).** Under basal conditions, no differences in the time spent in the open arms were observed between MS and control (animal facility reared, AFR) rats. One way ANOVA with Bonferroni *Post hoc* test, showed that WD 24 h induced a significant reduction in the time spent in the open arm in both AFR and MS groups (^*^^*^^*^p < 0.001 for WD (24 h) compared to basal, for each of the two groups AFR and MS). Reduction in time spent in open arms following WD (24 h) was significantly greater in the MS compared to the AFR group (^*^^*^^*^p < 0.001).

Anxiety was assessed using the EPM. To evaluate whether up-regulation of the VPMNN system could influence anxiety as measured in the EPM test, and if MS, which had resulted in a hyper-innervation pattern of AVP in the amygdala, could further influence anxiety in adults, we performed this behavior test with the following four groups: AFR basal, MS basal, AFR with 24 h of WD (AFR WD 24 h) and MS with 24 h of WD (MS WD 24 h; Figure 6). One-way ANOVA showed that the differences between means were statistically significant (*p* < 0.001, *F*_(3,36)_ = 26.44). *Post hoc* Bonferroni’s multiple comparisons test showed that under basal conditions no significant difference was seen between the percentage of time spent in the open arms by the AFR (29.75% ± 3.51%) and MS (29.13% ± 2.65%) rats (*p* > 0.05). WD significantly decreased the time spent in the open arms in both groups (*p* < 0.001, when compared with the corresponding basal condition) with WD 24 h reducing open arm time to 14.08% ± 2.45% in the AFR group, and to 4.67% ± 0.93% in the MS group. There was a significantly greater reduction in time spent in open arm following WD 24 h in the MS, than in the AFR group (*p* < 0.001).

### V1A Receptor mRNA Expression in Anterior and Central Amygdala

**Figure 7:**
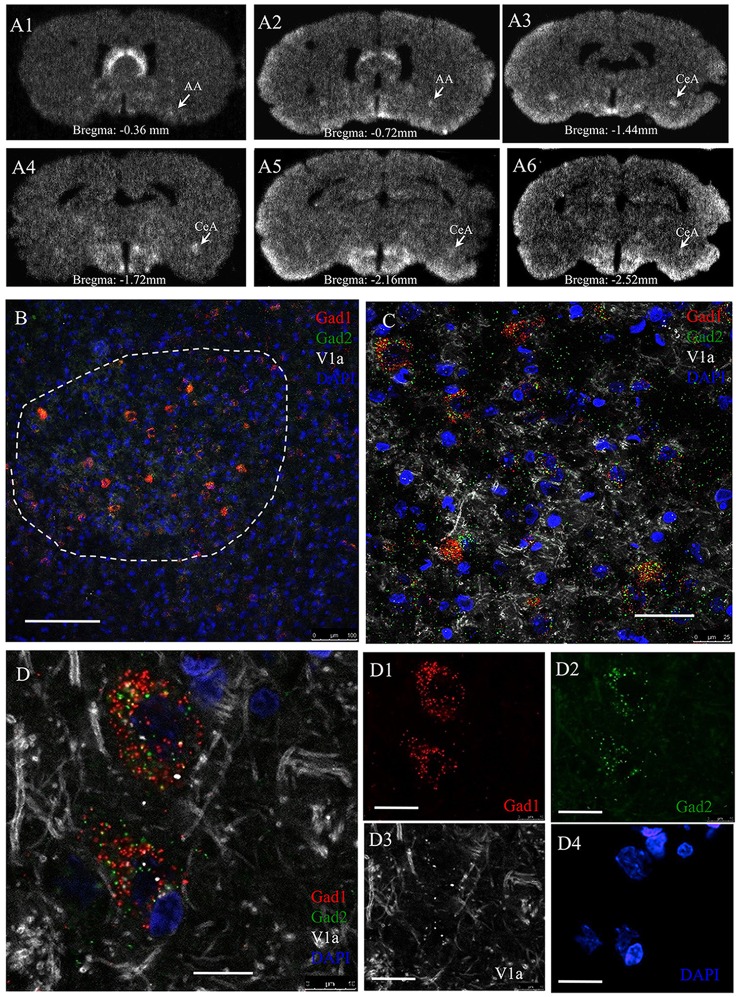
**AVP receptor V1a mRNA expression in GABAergic neurons in the CeA.** Panel **(A’s)** sequential coronal sections were hybridized with antisense radioactive AVP V1a riboprobe. Autoradiographs with overnight-exposure were read by phosphorimager. Rostro-caudal coordinates were indicated according to Rat Brain Atlas (Paxinos and Watson, 2006). AA, Anterior amygdala; CeA, central amygdala. **(B–D)** *In situ* hybridization using RNAscope-multiplex method targeting the Gad-1, Gad-2 and V1a mRNAs in CeA. V1a mRNA is particularly expressed by Gad-1 and Gad-2 positive neurons. **(B)** Low magnification showing the selective location of Gad1 + Gad2 expressing cell population in the CeA (dash-line circumscribed region). **(C)** Further magnifications showing the exclusive expression of V1a mRNA in Gad1 and Gad2 positive cells (100%, *n* = 98 neurons analyzed). Optical section thickness 1 μm. **(D’s)** Confocal 1 μm optical slice of two cells in CeA expressing Gad1 (red), Gad2 (green) and V1a receptor (white) mRNA. Using the same method for V1b mRNA, we did not find any positive labeling. This data is not shown. The positive control for this latter experiment was done using adenohypophysis tissue. Scale bars: **(B)** 100 mm; **(C)** 20 μm; **(D)** 10 μm.

V1a receptor mRNA is strongly expressed in AA and CeA while V1b and V2 mRNA were not detectable using the RNAscope method. The V1a mRNA expression had a complete co-localization with Gad1 and Gad2 mRNA in CeA. Based on the foregoing findings, we hypothesized that the some or all the currently identified GPCRs for vasopressin, namely the V1a, V1b and V2 receptors, were involved in the anxiogenic action of vasopressin during the WD 24 h. We first successfully developed a V1a riboprobe based on the procedures of Morel et al. (1992) and we used the traditional ISH method to examine the V1a mRNA in the whole amygdaloidal complex (including rostro-caudal span). Phospho-imager detection showed strong and localized expression of V1a mRNA in the AA and CeA from Bregma −0.36 mm to –2.52 mm (Figures 7As). To confirm this finding, we used another highly-sensitive RNAscope ISH method, which allowed us to simultaneously detect V1a mRNA together with Gad1 and Gad2 mRNA in CeA. Figures 7B–D’s shows representative confocal images under low and high magnification, illustrating the complete co-localization of V1a mRNA with the Gad1 and Gad2 mRNA. V1b and V2 probes for RNAscope, which gave positive signals in anterior pituitary gland and renal tissues, did not yield any positive labeling in the amygdaloidal complex (data not shown).

**Figure 8:**
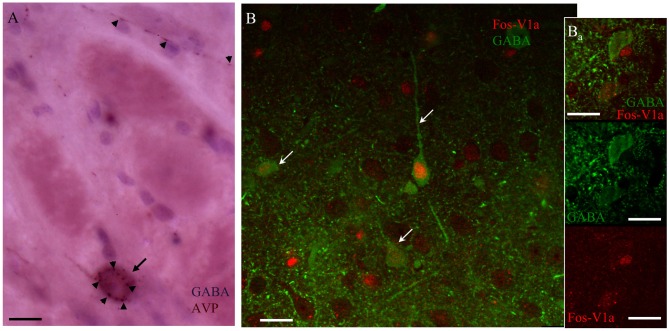
**AVP containing fibers in CeA synapse upon V1a receptor-positive GABAergic neurons activated after EPM. (A)** Immunohistochemistry against AVP (arrowheads) and GABA (arrow) showing punctate AVP fibers terminals in close apposition with the GABA-labeled soma. **(B)** Confocal image of CeA immunoreacted against FOS (red nuclei indicated by arrows); V1a receptor (red punctuated label, indicated by arrowheads) and GABA (green cells). The immunohistochemistry was performed 90 min after the EPM in an animal infused with vasopressin in the CeA. **(B_a_)** A different optical section of the same region showing two GABA+ neurons, one of them negative for V1a receptor showing no expression of Fos, the other one expressing the V1a receptor being Fos+. Scale bars: 10 μm.

Using immunohistochemistry, we observed AVPir+ fibers surrounding the GABAir+ soma in the CeA (Figure 8A). Figure 8Bs shows an example taken from CeA of a rat perfused 90 min after the EPM test from the group of MS/WD 24 h, where all the GABAir+ neurons exhibited nuclear Fos expression, a marker for neuronal activation, and also had membrane V1a labeling.

### AVP Infusion into CeA Induced Anxiogenesis via a V1aR Pathway

**Figure 9:**
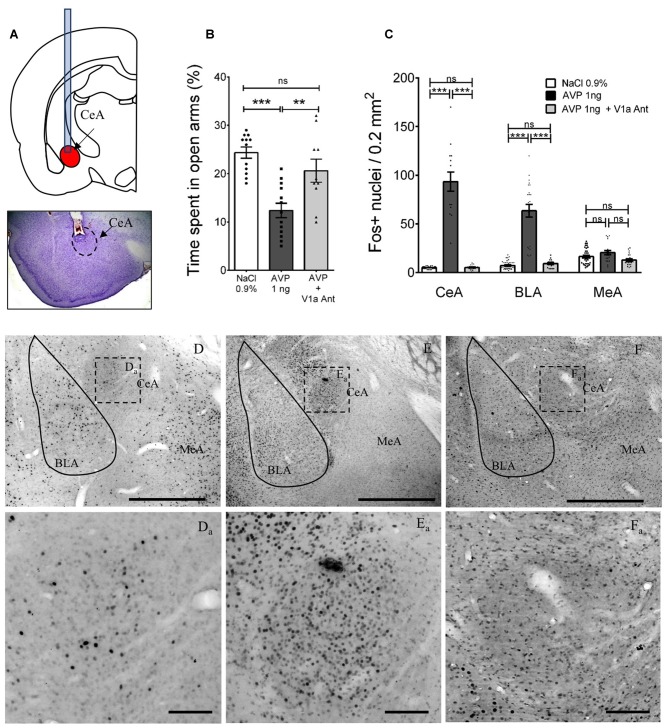
**Altered anxious behavior and Fos expression after AVP infusion into CeA in AFR rats and reversal by co-infusion of V1a antagonist (Manning compound). (A)** Upper panel depicts positioning of cannulae in CeA for infusion of vehicle, AVP, or AVP plus V1a antagonist (Manning compound); lower panel is a representative photomicrograph showing the trajectory of the cannula toward the CeA. **(B)** Bar graph showing the time spent in the open arms of the EPM in animals infused bilaterally with vehicle (NaCl 0.9%), AVP or AVP + V1a antagonist (Manning compound). Infusion of AVP into CeA produced anxiety-like behavior shown as a reduction of the time spent in the open arms (^*^^*^p < 0.01, ^*^^*^^*^p < 0.001) compared with vehicle-treated rats. Infusion of AVP + V1a antagonist reversed anxious behavior (decreased time spent in open arms) to control levels (i.e., indistinguishable from vehicle-treated rats). **(C)** Bar graph showing the quantification of neurons activated (measured by Fos expression) after EPM in the CeA, BLA and MeA in vehicle, AVP, or AVP plus V1a antagonist infused groups. AVP produced a significant increase in the Fos expression in the central (CeA, *p* < 0.001) and basolateral nuclei (BLA, *p* < 0.001) that was blunted in the AVP + V1a antagonist group. No effect of the treatments was observed in the MeA. **(D–F)** Photomicrographs showing the Fos expression patterns seen in the three infusion groups after the EPM, **(D_a_–F_a_)** are magnifications of the squared regions in **(D–F)**. BLA, basolateral amygdala; CeA, central amygdala; MeA, medial amygdala. Scale Bars: **(D–F)** 1 mm; **(D_a_–F_a_)** 0.1 mm.

To further evaluate the effect on anxiety of increased vasopressin levels in amygdala, vasopressin (1 ng), vasopressin ((1 ng) + the vasopressin V1a receptor antagonist Manning compound (10 ng)) or vehicle (NaCl 0.9%) was infused at each of two sites 15 min before the EPM test, via cannulas stereotaxically placed in the CeA (Figure 9A). One-way ANOVA showed that the differences between means were statistically significant (*p* < 0.0001, *F*_(2,32)_ = 15). *Post hoc* Bonferroni’s multiple comparisons test showed that vasopressin infusion significantly (*p* < 0.001) diminished the percentage of time spent in the open arms (12.38% ± 1.48%) with respect to the vehicle group (24.33% ± 1.16%). The AVP + V1a antagonist group showed a significant increase in this value (29.75% ± 3.51%) with respect to the AVP group (*p* < 0.01) and was no different than the vehicle treated group (Figure 9B).

### Vasopressin Infusion Enhanced the Neuronal Activation in CeA and BLA After the EPM

To evaluate the effect of the infusion of vasopressin, vasopressin + the V1a receptor antagonist (Manning compound) or vehicle in the number of neurons activated by the EPM in the CeA, BLA and MeA, we perfused the rats 90 min after finishing the EPM test, sliced the brains, and performed immunohistochemistry against c-Fos. Five brains, in which correct cannula placement in CeA was confirmed, were selected to count the number of Fos+ nuclei in an area of 0.2 mm^2^, inside the CeA, BLA and MeA in 5–6 slices around the injection site (Figure 9C). Panels (D–F) and inserts of Figure 9 show examples of these immunoreactions in the vehicle, AVP and AVP + V1a antagonist treated rats.

Using a two-way ANOVA analysis (factors: region and treatment), we observed a significant effect for region: *F*_(2,225)_ = 28.9, *p* < 0.0001; treatment: *F*_(2,225)_ = 285.5, *p* < 0.001 and their interaction: *F*_(4,225)_ = 67.2, *p* < 0.001. Tukey’s multiple comparisons test showed significant differences in the CeA between the vehicle and AVP infused groups: *F*_(2,225)_ = 27.95, *p* < 0.001 and between the AVP and AVP + V1a antagonist: *F*_(2,225)_ = 24.68, *p* < 0.001; no difference between vehicle and AVP + V1a antagonist was found: *F*_(2,225)_ = 0.035, *p* > 0.05. In the BLA region a similar pattern was found, with significant differences between the vehicle and AVP infused groups: *F*_(2,225)_ = 21.26, *p* < 0.001 and between the AVP and AVP + V1a antagonist: *F*_(2,225)_ = 18.07, *p* < 0.001; no difference between vehicle and AVP + V1a antagonist was found: *F*_(2,225)_ = 0.75, *p* > 0.05. With respect to the MeA, no significant differences were found between any of the treatment groups.

## Discussion

In this study, we investigated vasopressinergic innervation of the rat amygdaloid complex, and its functional implications. We quantified the density of AVP innervation in the subnuclei of the amygdaloid complex and demonstrated that in parallel to the AVP containing neurons in the STIA, previously described (Caffé and van Leeuwen, 1983; Plumari et al., 2002; Rood and De Vries, 2011), which are likely to be GABAergic neurons (Dabrowska et al., 2013), there exist a direct AVP-containing glutamatergic pathway, from VPMNN axon collaterals, synaptically targeting the CeA. We report that early life stress (MS) increased the density, and altered the pattern, of AVP innervation to amygdala in agreement with the enlarged PVN and SON previously reported (Zhang et al., 2012). In CeA, mRNA encoding the V1a receptor was detected as highly expressed and, using the newly developed RNAscope method, we could demonstrate that all the cells expressing V1a mRNA in CeA were Gad1- and Gad2-expressing cells. Using immunohistochemistry, we observed that GABAergic neurons inside the CeA expressed V1a receptor protein on their plasma membrane. Physiological up-regulation of the VPMNN system by WD 24 was associated with higher expression of anxiety-like behavior, and increased Fos expression in the CeA, mainly within GABA-positive neurons. The effects of WD 24 were mimicked by AVP local micro-infusion into the CeA, and these effects, and those of WD 24 itself, were attenuated by local infusion of Manning compound a V1a receptor specific antagonist-into the CeA.

A significant body of literature implicates AVP as a transmitter subserving stress responses in adult rodents without a history of stressor exposure, and in adult rodents with a history of stressor exposure during early post-natal life (Veenema and Neumann, 2008; Zhang et al., 2010, 2012, 2016; Zhang and Hernández, 2013; Hernández et al., 2015). A potential linkage between these two AVP-related types of stress response is found in the observations that early-life stress, in rats, leads to reorganization of vasopressinergic neurons of the PVN (Zhang et al., 2012). However, an anatomical connection between effects of AVP on behavioral responses to stress, mainly associated with AVP receptors resident in the amygdala, and specific projections from paraventricular VPMNNs, has not yet been made. As we have previously identified axonal projections from VPMNNs to various extrahypothalamic brain regions (Hernández et al., 2015), including those putatively involved in stress responding (Zhang and Hernández, 2013; Zhang et al., 2016), we hypothesized that VPMNN projections to the amygdala might modulate both adult stress/anxiogenic circuits, and provide an anatomical substrate for adult stress response conditioning occurring during the neonatal period. Accordingly, we designed and carried out a functional neuroanatomical study of AVP VPMNNs projections to amygdala, their putative neurons of origin, and the potential role of these AVP neurons in stress response modulation in both acute adult, and developmentally determined, behavior contexts.

In brief (*vida supra*), our data suggest that vasopressinergic projections, originating from magnocellular soma of the PVN (VPMNNs), synapse upon GABAergic neurons of the CeA which contain V1a receptors; that the CeA projections of these neurons are increased by early-life stress (MS); that activation of these neurons via WD in adulthood is anxiogenic; that the degree of anxiogenesis is correlated with the number and AVP content of these projections; and that anxiogenesis correlated with the dynamic changes of VPMNN system projecting to the CeA can be mimicked by CeA infusion of AVP, and blocked by CeA infusion of a V1a antagonist. What are the implications of these findings?

First, the localization of AVP-dependent stress responding to VPMNNs of the PVN, in both adult stress responding and in developmental shaping of the stress response, allows the testing of further important hypotheses about the amygdalar microcircuitry of stress, and the extent of AVP involvement in this circuitry. Thus, we may now hypothesize that the locus of action, or at least one anatomical target, of psychogenic stressors such as MS in early life, that affect long-term neuroplasticity of the VPMNN population, is at synapses on GABAergic neurons of the CeA. It is noteworthy that MS does not cause increased adult anxious behavior on its own, but rather increases the penetrance of other stressors (thirst, this study; electrical shock in previous studies—see Zhang et al., 2012) in a way that requires AVP transmission within the CeA. Examination of the corresponding dependence on AVP release within the CeA, as probed with local infusion of V1A antagonist, of other adult and anxiogenic stressors, including restraint, social defeat, and social subordination, appears highly warranted by the results presented here.

Second, the localization to GABAergic neurons of CeA of these vasopressinergic effects on stress responding raises the important question of where these circuits ultimately find expression in altered exploratory and other anxiety-associated behaviors in the rodent. The recent expansion of detailed knowledge about GABAergic neurons in CeA involved in mediating the salience of negative (fear- and anxiety-inducing) environmental stimuli now allows more anatomically detailed inquiry into the precise role of VPMNN projections in this circuitry (for example see Haubensak et al., 2010).

Finally, this work exposes a new opportunity to test the relative specificities of various neuropeptides in modulation of the stress response. The metabolic activation of VPMNNs following 24 h of thirst is itself a stressor, but a very specific paraphysiological one, that has allowed a clear linkage between VPMNN activation and anxiogenesis to be revealed (vide supra). However this work also allows the testing of AVP and VPMNN dependence of behavioral responses to other stressors, as well as exploring whether or not AVP release onto GABAergic synapses in CeA is a general feature of amygdala-mediated modulation of emotional responses to stress, or is reserved to specific subtypes of stress responding, with other neuropeptides mediating different and equally specific modes of stress response modulation.

## Author Contributions

LZ and VSH: conceived and designed the experiments. LZ, VSH, ORH and MJG: performed the experiments. LZ, VSH, ORH, MPM, KF and LEE: analyzed the data. LZ, LEE and MPM: contributed equipment/reagents/materials/analysis tools. LZ, VSH and LEE: wrote the article. VSH, ORH, MPM, MJG, KF, LEE and LZ: revised the manuscript critically for important intellectual content.

## Conflict of Interest Statement

The authors declare that the research was conducted in the absence of any commercial or financial relationships that could be construed as a potential conflict of interest. The reviewer MO and handling Editor declared their shared affiliation, and the handling Editor states that the process nevertheless met the standards of a fair and objective review.
