# Supplementary material for: Hypothalamic Vasopressinergic Projections Innervate Central Amygdala GABAergic Neurons: Implications for Anxiety and Stress Coping
Source: Front Neural Circuits. 2016 Nov 18;10:92. doi: 10.3389/fncir.2016.00092 (PMC5122712; doi:10.3389/fncir.2016.00092)
Supplement: Supplementary file 1 [file Table_1.docx]

| Molecule | Host species | Dilution | Source | Source code | Antibody Specificity Information |
| --- | --- | --- | --- | --- | --- |
| [Arg8 ]-vasopressin | Rabbit | 1:5000 | Peninsula-Bachem Americas, Inc., CA, USA. (www.bachem.com) | T-4563 | [[1](#_ENREF_1), [2](#_ENREF_2)] |
| [Arg8 ]-vasopressin | Rabbit | 1:2000 | Prof. R.M. Buijs, Instituto de Investigaciones Biomédicas, Universidad Nacional Autónoma de México, UNAM | -- | [[3](#_ENREF_3)] |
| c-Fos | Rabbit | 1:2000 | Santa Cruz Biotechnology, Dallas, Texas U.S.A (www.scbt.com) | SC-52 | [[4-7](#_ENREF_4)] |
| Vesicular glutamate transporter 2 | Guinea pig | 1:1000 | Frontier Institute Co., Ltd., Hokkaido , Japan (www.frontier-institute.com) | GP-AF240-1 | [[8](#_ENREF_8), [9](#_ENREF_9)] |
| Gamma-aminobutyric Acid (GABA) | Mouse | 1:1000 | Sigma-Aldrich Corporation, MO, USA (www.sigmaaldrich.com) | A0310 | [[10-12](#_ENREF_10)] |
| Vasopressin V1A Receptor | Rabbit | 1:1000 | Alomone Labs. Jerusalem, Israel. (www.alomone.com/) | AVR-010 | Tested with western blot and blockade of antibody binding by contol peptide provided by the company |

**Table SI-1: Antibody information, related to Material and Method**

**References**

1. Taylor, A.C., J.J. McCarthy, and S.D. Stocker, *Mice lacking the transient receptor vanilloid potential 1 channel display normal thirst responses and central Fos activation to hypernatremia.* Am J Physiol Regul Integr Comp Physiol, 2008. **294**(4): p. R1285-93.

2. Zhang, L. and V.S. Hernandez, *Synaptic innervation to rat hippocampus by vasopressin-immuno-positive fibres from the hypothalamic supraoptic and paraventricular nuclei.* Neuroscience, 2013. **228**: p. 139-62.

3. Buijs, R., et al., *Antibodies to small transmitter molecules and peptides: production and application of antibodies to dopamine, serotonin, GABA, vasopressin, vasoactive intestinal peptide, neuropeptide Y, somatostatine and substance P.* Biomedical research, 1989. **10**(supplement 3): p. 213-221.

4. Leao, R.M., et al., *Chronic nicotine activates stress/reward-related brain regions and facilitates the transition to compulsive alcohol drinking.* J Neurosci, 2015. **35**(15): p. 6241-53.

5. Soga, T., et al., *Early-Life Social Isolation Impairs the Gonadotropin-Inhibitory Hormone Neuronal Activity and Serotonergic System in Male Rats.* Front Endocrinol (Lausanne), 2015. **6**: p. 172.

6. Velazquez, F.N., et al., *Brain development is impaired in c-fos -/- mice.* Oncotarget, 2015. **6**(19): p. 16883-901.

7. Zuloaga, D.G., et al., *Enhanced functional connectivity involving the ventromedial hypothalamus following methamphetamine exposure.* Front Neurosci, 2015. **9**: p. 326.

8. Miyazaki, T., et al., *Subtype switching of vesicular glutamate transporters at parallel fibre-Purkinje cell synapses in developing mouse cerebellum.* Eur J Neurosci, 2003. **17**(12): p. 2563-72.

9. Zhang, S., et al., *Dopaminergic and glutamatergic microdomains in a subset of rodent mesoaccumbens axons.* Nat Neurosci, 2015. **18**(3): p. 386-92.

10. Gonchar, Y., Q. Wang, and A. Burkhalter, *Multiple distinct subtypes of GABAergic neurons in mouse visual cortex identified by triple immunostaining.* Front Neuroanat, 2007. **1**: p. 3.

11. Omelchenko, N. and S.R. Sesack, *Cholinergic axons in the rat ventral tegmental area synapse preferentially onto mesoaccumbens dopamine neurons.* J Comp Neurol, 2006. **494**(6): p. 863-75.

12. Sloviter, R.S., et al., *Substance P receptor expression by inhibitory interneurons of the rat hippocampus: enhanced detection using improved immunocytochemical methods for the preservation and colocalization of GABA and other neuronal markers.* J Comp Neurol, 2001. **430**(3): p. 283-305.
